# Explainable Artificial Intelligence Reveals Novel Insight into Tumor Microenvironment Conditions Linked with Better Prognosis in Patients with Breast Cancer

**DOI:** 10.3390/cancers13143450

**Published:** 2021-07-09

**Authors:** Debaditya Chakraborty, Cristina Ivan, Paola Amero, Maliha Khan, Cristian Rodriguez-Aguayo, Hakan Başağaoğlu, Gabriel Lopez-Berestein

**Affiliations:** 1 Department of Construction Science, The University of Texas at San Antonio, San Antonio, TX 78249, USA; 2 Department of Experimental Therapeutics, The University of Texas MD Anderson Cancer Center, Houston, TX 77030, USA; CIvan@mdanderson.org (C.I.); pamero@mdanderson.org (P.A.); CRodriguez2@mdanderson.org (C.R.-A.); glopez@mdanderson.org (G.L.-B.); 3 Center for RNA Interference and Non-Coding RNA, The University of Texas MD Anderson Cancer Center, Houston, TX 77030, USA; 4 Department of Lymphoma and Myeloma, The University of Texas MD Anderson Cancer Center, Houston, TX 77030, USA; MKhan4@mdanderson.org; 5 Evolution Online LLC, San Antonio, TX 78260, USA; hakanbasagaoglu@hotmail.com

**Keywords:** explainable artificial intelligence (XAI), machine learning, breast cancer, tumor microenvironment, survival analysis

## Abstract

**Simple Summary:**

Over the past decade, there has been a significant increase in the number of omics datasets that provide unprecedented opportunities to systematically characterize the underlying biological mechanisms involved in cancer evolution and to understand how the tumor microenvironment contributes to this evolution. Novel techniques in artificial intelligence (AI) can help determine areas of therapeutic need, enhance clinical trial interpretation, identify novel targets, and generate accurate predictions that are impossible with traditional statistical techniques. However, a major criticism of incorporating the highly accurate and nonlinear AI models into medical fields is the notion that AI is essentially a “black box”. We resolved this overarching problem with explainable artificial intelligence (XAI) to determine prognoses in patients with breast cancer and reveal valuable information about conditions in the tumor microenvironment that are associated with enhanced prognosis and patient survival. The benefits of using XAI in the development of new targeted therapies would be significant.

**Abstract:**

We investigated the data-driven relationship between immune cell composition in the tumor microenvironment (TME) and the ≥5-year survival rates of breast cancer patients using explainable artificial intelligence (XAI) models. We acquired TCGA breast invasive carcinoma data from the cbioPortal and retrieved immune cell composition estimates from bulk RNA sequencing data from TIMER2.0 based on EPIC, CIBERSORT, TIMER, and xCell computational methods. Novel insights derived from our XAI model showed that B cells, CD8^+^ T cells, M0 macrophages, and NK T cells are the most critical TME features for enhanced prognosis of breast cancer patients. Our XAI model also revealed the inflection points of these critical TME features, above or below which ≥5-year survival rates improve. Subsequently, we ascertained the conditional probabilities of ≥5-year survival under specific conditions inferred from the inflection points. In particular, the XAI models revealed that the B cell fraction (relative to all cells in a sample) exceeding 0.025, M0 macrophage fraction (relative to the total immune cell content) below 0.05, and NK T cell and CD8^+^ T cell fractions (based on cancer type-specific arbitrary units) above 0.075 and 0.25, respectively, in the TME could enhance the ≥5-year survival in breast cancer patients. The findings could lead to accurate clinical predictions and enhanced immunotherapies, and to the design of innovative strategies to reprogram the breast TME.

## 1. Introduction

Breast cancer is the most common cancer and the leading cause of cancer death in women worldwide. The prognosis is dependent on the type of breast cancer and on the stage of disease at detection [1,2]. Breast cancer can be divided into several subtypes, based primarily on the expression of estrogen receptor (ER), progesterone receptor (PR), and human epidermal growth factor receptor 2 (HER2). Triple-negative breast cancer (TNBC) is a heterogeneous category of breast cancer, characterized by negative ER, PR, and HER2. TNBC is highly metastatic and aggressive, with poor prognosis, poor patient survival, and limited therapeutic options [3].

Cancer development, progression, and treatment resistance are known to be influenced by genetic and epigenetic alterations as well as by crosstalk between tumor cells and the tumor microenvironment (TME) [4]. The TME involves a complex network of soluble factors, tumor cells, and stromal cells that plays a crucial role in the initiation, development, and progression of breast cancer. The TME in breast cancer consists of multilineage immune cells (e.g., T and B lymphocytes, myeloid cells, and dendritic cells), cancer-associated fibroblasts, and tumor endothelial cells [5]. These cell subtypes live in an ocean of hormones, growth factors, and cytokines in the breast TME [6]. Adding to this complexity is a myriad of pathways that dictate the fate of tumor, metastases, and patients’ lives. The balance between tumor-infiltrating immune effector cells in the TME (such as CD4^+^ T cells and CD8^+^ T cells (or cytotoxic T lymphocytes, CTLs)) regulates the immune response to cytotoxic effects on tumor cells. In a mouse model of metastatic breast cancer, natural killer (NK) T cell activation was shown to enhance antitumor immunity by increasing cytotoxic responses and interferon-γ production from NK and CD8^+^ T cells [7]. In contrast, tumor-infiltrating myeloid cells, such as tumor-associated macrophages, promote the expansion and dissemination of cancer cells depending on their functional state [8]. Hypoxia in the TME stimulates macrophages to further produce proangiogenic factors such VEGF and suppress the T cell immune responses, hence enhancing the evasion of tumor cells and ultimately metastasis [8]. TME crosstalk potentially promotes cancer progression and TME plasticity. Adaptations to TME factors may be responsible for metastasis and immune evasion.

Multiple targeted therapies have been used for TNBC but to no avail. Aberrant signaling by VEGFR2 and cMET, as well as modifications of the immune cell population in response to an immune-suppressive phenotype, resulted in a failure of immunotherapy for TNBC [9]. In TNBC, multiple genomic instabilities and mutations have been associated with immune responses [10]. A comparative study between TNBC and non-TNBC (NTNBC) showed that TNBC is characterized by higher expression levels of functional gene sets associated with 15 types of immune cells [11,12]. Unfortunately, innovative strategies to reprogram the TME of breast cancer patients is challenging, in part because of conflicting findings in the literature. For example, tumor-infiltrating B lymphocytes (B cells) have been reported to be associated with positive, negative, or no significance in breast cancer prediction and prognosis [13]. Other studies have reported that targeting the regulatory B cell activity may be used to enhance immunotherapeutic outcomes [14].

Augmentation of CTL-induced antitumor immune reactions has been considered to be an attractive therapeutic modality for lethal solid tumors due to the tumor-killing ability of CD8^+^ CTL [15]. In the adaptive immune system, T-helper cells (CD4^+^ cells) play a critical role in releasing cytokines and primarily help CD8^+^ CTL and antibody responses to mediate antitumor immunity. However, the interplay between polyfunctional CD4^+^ T cells and other immune cell lineages within the context of tumor immunity is not well understood [16]. In addition to immune cells, tumor-associated factors in the TME have also been targeted in cancer therapies. The recognition that tumor-associated endothelial cells and cancer-associated fibroblasts are important mediators of immune suppression has led to the development of cell-specific targeting drugs in an attempt to enhance the immune response [5]. Tumor-associated macrophages are able to suppress the functions of CD8^+^ T and NK cells and promote tumor cell growth in the TME [17].

Over the past decade, there has been a major increase in the number of large and complex omics datasets [18,19], especially through large consortium projects such as TCGA, which has sampled multiomics measurements from more than 30,000 patients and dozens of cancer types [20]. These rich omics data provide unprecedented opportunities to systematically characterize the underlying biological mechanisms involved in the evolution of cancer and to understand how the TME (hosting stromal cells, immune cells, and other types of cells) contributes to this evolution [19,21].

Novel techniques in AI can bring together diverse data types to expand novel insights gained from the multiomics datasets. It is well acknowledged that enrichment of high-quality data coupled with machine learning, a subset of AI, can help investigate the areas of changing patients’ unhealthy behaviors [22], risk prediction or recurrence prediction of chronic diseases after a surgery [23] and curative treatment [24], progression and survivability of patients with chronic diseases [25], therapeutic need, enhanced clinical trial interpretation, and novel targets [26]. However, a major criticism of incorporating AI, particularly deep learning, into medical fields is the idea that AI is essentially a mechanistically uninterpretable opaque “black box” [27,28,29], and hence may not meet the required high level of accountability, transparency, and reliability in medical decisions [30]. The assumed lack of interpretability of AI models has been a debated topic within the field, with models cited that have achieved high accuracy due to factors that are not useful in prospective predictions [27,31,32]. The crux of the problem is that linear models, although interpretable, produce less accurate models when the datasets are complex and inherently nonlinear. In such cases, tree-based ensemble models, which are interpretable models [24], can be used in lieu of deep learning models that allow scientists and clinicians to understand the underlying reasoning behind the decisions and predictions. Recently, Gu et al. [33] used a tree-based ensemble model, called an extreme gradient boosting (XGBoost) model [34], to predict the risk of breast cancer relapse from clinical data (e.g., age, tumor size, treatment) and then use case-based reasoning—which solves new problems by constructing a historical case base and using the results of similar historical cases—to explain the reason for the prediction. In addition, the authors used a game theory-based Shapley additive explanation model called SHAP [35,36] for global explainability of the results to identify the order of importance of the clinical features considered.

In this article, we develop explainable artificial intelligence (XAI) models to establish and investigate the data-driven relationship between TME features, which comprise a vast variety of immune cells, and the ≥5-year survival rates of breast cancer patients. The XAI models also determine relative influence of immune cells (e.g., T cells, B cells) and tumor-associated cells (e.g., macrophages) in the TME on the ≥5-year survival rates of patients. In addition, using XAI models and conditional probabilities, we identified and analyzed the inflection points of the critical microenvironment features, above or below which the ≥5-year survival rates could potentially improve. The resulting new perspective on favorable or deleterious microenvironmental conditions could lead to improved prognoses through well-informed clinical management and therapeutics, including the design of innovative strategies to reprogram the TME of breast cancer patients.

## 2. Materials and Methods

We downloaded patient clinical information for TCGA breast-invasive carcinoma cohort (BRCA) from two projects on the cbioPortal (http://www.cbioportal.org/) (accessed on 1 February 2021) [37,38]—the PanCanAtlas [20,21]—and the Firehose Legacy (https://gdac.broadinstitute.org/) (accessed on 1 February 2021) that provided clinical information for 1101 patients. We found 1015 patients with primary tumor samples common to both projects. For these 1015 primary tumors, we searched TIMER2.0 (http://timer.cistrome.org/) (accessed on 1 February 2021) for immune infiltration estimations produced with EPIC [39], CIBERSORT [40], TIMER [41], and xCell [42] computational methods [43]. We ended up with a cohort of 1014 breast cancer patients with clinical information and estimates of immune cell content in tumor tissues.

Subsequently, we developed data-driven XAI models using XGBoost and SHAP to enhance the explainability of the breast cancer survivability models based on TME conditions (including both immune cells and tumor-associated cells), understand the underlying reasoning, and expand our knowledge without compromising the predictive accuracy. Moreover, in addition to the global SHAP analysis to determine the order of importance of the TME cells on the patients’ survivability rates, we performed local SHAP analysis to identify the inflection points for the TME cells, above or below which the survivability rates may increase. We demonstrated that the local SHAP analysis expanded the potential use of the interpretable AI model to investigate potential immunotherapies to increase patients’ survivability rates with enhanced explainability and transparent reasonings.

XGBoost is a variant of a tree-based boosting algorithm. Conceptually, XGBoost learns the functional relationship f between the features x and target y through an iterative process in which the individual trees are sequentially trained on the residuals from the previous tree. Mathematically, the predictions from the trees can be expressed as

(1) $$\hat{y}=\phi \left(x\right)=\frac{1}{n}\;\overset{n}{\underset{k=1}{\sum }}f_{k}\left(x\right)$$

where $\hat{y}$ is the predicted outcome (overall survival and 5-year survival) in breast cancer patients, 1≤k≤n, and $f_{1},\;f_{2},\ldots ,\;f_{n}$ are the functions learned by n number of trees.

The following regularized objective ℒ (ϕ) is minimized to learn the set of functions $f_{k}$ used in the model

(2) $$ℒ\;\left(\phi \right)=\underset{i}{\sum }l\left(\hat{y}-y\right)+\underset{k}{\sum }\Omega \left(f_{k}\right)$$

where $\Omega \left(f_{k}\right)=\gamma T+\frac{1}{2}\;\lambda {\left|\left|w\right|\right|}^{2}$.

In Equation (2), l is the differentiable convex loss function that measures the difference between $\hat{y_{i}}$ and $y_{i}.\;\Omega$ is an extra regularization term that penalizes the growth of more trees in the model to prevent complexity, and thus reduce overfitting. γ is the complexity of each leaf, T is the number of leaves in a tree, λ is a penalty parameter, and ||w|| is the vector of scores on the leaves. Note that if the regularization parameter Ω is set to zero, the objective falls back to the traditional gradient tree boosting.

SHAP, in contrast, was used to explain the AI models by investigating the relationship and contribution of each feature to the predicted AI-based outcome ($\hat{y}$). SHAP computes the Shapley values that signify the average marginal contribution of each feature value across all possible combinations of features. The features with large absolute Shapley values are deemed impactful. To evaluate the overall feature influence on the predicted outcome, SHAP averages the absolute Shapley values for every feature across the data, sorts them in decreasing order, and plots them. In our work, negative Shapley values associated with the feature instances indicate better chances of ≥5-year survival.

## 3. Results

**Figure 1:**
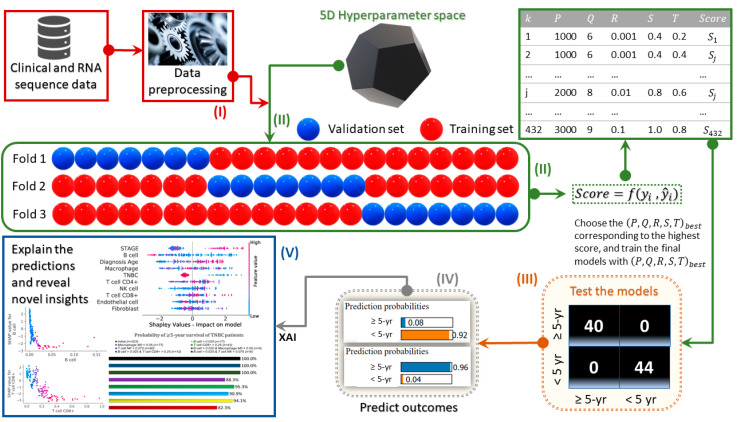
Schematic representation of the modeling pipeline: (I) data preprocessing steps, which include encoding categorical features as integer arrays and data oversampling to convert from an imbalanced to a balanced dataset (i.e., the numbers of surviving and deceased patients were made nearly equal) to avoid bias (i.e., preventing the model from ignoring minority classes); (II) hyperparameter optimization via a 3-fold cross-validation to find the best subset of hyperparameters (k: hyperparameter subset index number, P: number of estimators, Q: maximum depth of each estimator, R: learning rate, S: subsample ratio, T: column sample ratio for each estimator) that improves the models’ ROC–AUC score {$f(y_{i},{\hat{y}}_{i}$)} signifying the area under the receiver operating characteristic curve during the (432 × 3) iterations over the hyperparameter space (i.e., to enhance the predictive accuracy of the XAI models); (III) testing of the predictive accuracy of the final AI models after being trained with the best subset of hyperparameters; (IV) prediction of the probability of the clinical outcomes with AI models; (V) explanation of the predicted outcomes with a game theory-based XAI model to enhance the interpretability and explainability of the model predictions, identification of critical inflection (turning) points, above or below which the ≥5-year survival rates increase, and assessing the conditional probability of ≥5-year survival rates from the range of TME factors determined by the inflection points.

We developed models with the XAI pipeline, shown schematically in Figure 1, to predict the probability of ≥5-year survival of breast cancer patients based on immune cell composition from bulk RNA sequencing (RNA-seq) data estimated using EPIC, CIBERSORT, TIMER, and xCell methods. The XAI models were developed through a multistep process shown in Figure 1, which includes: (I) data preprocessing steps, (II) hyperparameter optimization via a three-fold cross-validation to find the best subset of hyperparameters, (III) testing of the predictive accuracy of the final AI models after being trained with the best subset of hyperparameters, (IV) prediction of the probability of the clinical outcomes, and (V) explanation of the predicted outcomes with a game theory-based XAI model to reveal the contributing factors and respective values of the TME constituents associated with better outcomes (≥5 years of survival) for breast cancer patients. Finally, we quantified the conditional probability of ≥5-year survival ($S_{5}$) for a given certain TME condition (C) using

(3) $$P(S_{5}|C)=100\times \frac{P\left(S_{5}\;\cap^{\;}\;C\right)}{P\left(C\right)}$$

where $P\left(S_{5}\cap^{\;}C\right)$ is the probability that both events $S_{5}$ and C occur simultaneously, and P(C) is the probability of condition (C) to occur.

**Figure 2:**
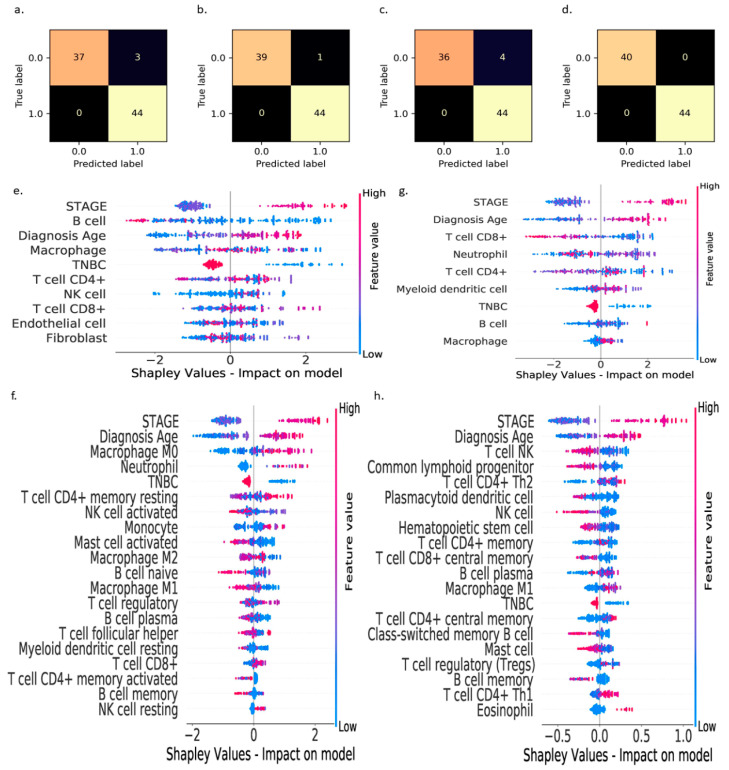
Predictive accuracies of our custom XAI models in terms of their ability to predict the likelihood of ≥5-year survival of breast cancer patients based on estimated immune cell composition from bulk RNA-seq data using EPIC (**a**), CIBERSORT (**b**), TIMER (**c**), and xCell (**d**) cell type quantification methods. The XAI indicates that the B cells (**e**), M0 macrophages (**f**), CD8^+^ T cells (**g**), and NK T cells (**h**) (estimated using EPIC, CIBERSORT, TIMER, and xCell methods, respectively) are the most important immune cells in the TME features in predicting survivability of breast cancer patients. The features on the y-axis are used in the respective models; their relative positions were determined by their relative importance in making correct predictions. The blue dots represent lower feature values, and the red dots represent higher feature values. In these analyses, Shapley values < 0 represent “likely to survive longer than 5 years after diagnosis” while Shapley values > 0 represent “likely to die within 5 years” after diagnosis.

With any data-driven XAI model, it is imperative to ensure that the model produces accurate predictions on testing samples that are not used during model training, and the prediction accuracies obtained during model training and testing are comparable to avoid overfitting or underfitting the data. We analyzed and reported the confusion matrices (Figure 2a–d) to gain a better understanding of the models’ performance in predicting the ≥5-year survival on the testing data that constitute 25% of the original dataset randomly sampled from the entire preprocessed dataset. These confusion matrices show that our AI models developed with the proposed pipeline produce reliable predictions on the testing data (Figure 2a–d). The accuracy, precision, recall, and F1 score of the proposed AI models on the testing data are tabularized in Table 1. These results indicate that the custom AI models are capable of accurately predicting (over 90%) the outcome for breast cancer patients based on the data associated with TME conditions in tumors.

After evaluating the predictive ability of the models, we used XAI to interpret our AI models’ predictions, investigate novel relationships between the TME features and the ≥5-year survival status, and identify the critical inflection points, above or below which the ≥5-year survival rates improve. We explain the custom XAI models from both global (entire dataset) and local (individual data points) perspectives. The global explanations revealed that the B cells, CD8^+^ T cells, M0 macrophages, and NK T cells are the most influential TME factors resulting from the RNA-seq data produced by the EPIC, CIBERSORT, TIMER, and xCell methods in determining the ≥5-year survival (Figure 2e–h). The XAI models suggest that higher CD8^+^ T cell, NK T cell, and B cell counts, along with low M0 macrophage count, lead to higher survival rates for breast cancer patients. It is worth noting that because different TME features are estimated by the EPIC, CIBERSORT, TIMER, and xCell methods, the relative importance of the TME features varied in the global SHAP analysis for each XAI model in Figure 2e–h.

The EPIC-XAI analysis (Figure 2e) revealed that B cells (antibody-producing machines) play a more critical role than other TME factors in improving the ≥5-year survival rates for breast cancer patients. Although the role of B cells was reported to be controversial in cancer immunotherapies [13], Figure 2e reveals that the presence and activation of larger numbers of B cells in the TME could enhance patients’ survivability rates and the efficacy of cancer immunotherapies. These findings are particularly important if the cancer is not at an advanced stage, at which a patient would be at higher risk of death according to Shapley analysis in Figure 2e–h. On the other hand, the CIBERSORT-XAI (Figure 2f) analysis revealed that M0 macrophages are the most influential TME factor that lowers the ≥5-year survival rates when present in larger numbers. CD8^+^ T cells and NK T cells were identified as the most critical tumor-suppressing immune cells by the TIMER-XAI and xCell-XAI analyses, respectively, for the enhanced ≥5-year survivability of the patients. We also found that the CD4^+^ T cells were identified by the XAI as the third most influential immune cell type on the breast cancer prognosis. These new findings and insights indicate an urgent need to rethink the current cancer immunotherapies that are largely focused on harnessing the antitumor CD8^+^ cytotoxic T cell response [12,16].

**Figure 3:**
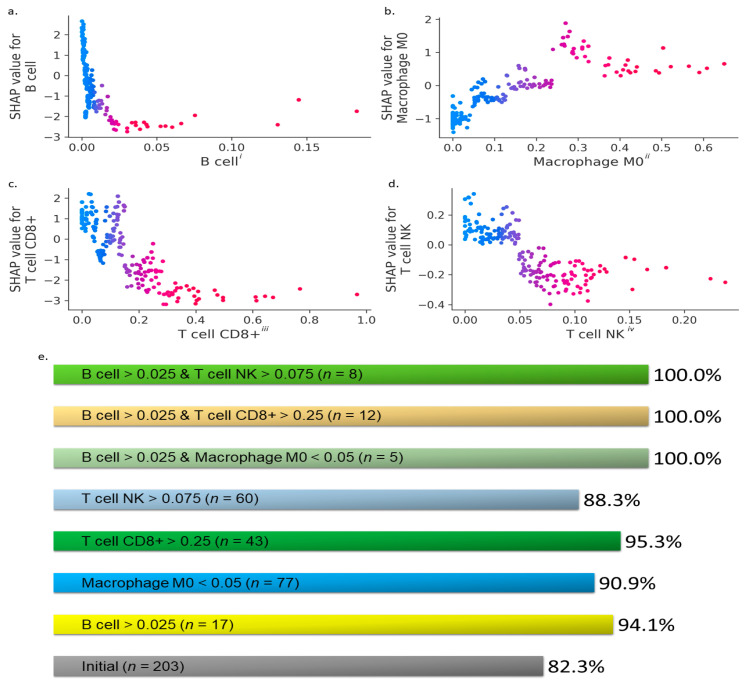
XAI results based on data from breast cancer patients who survived ≥5 years and are still alive, are dead after surviving ≥5 years, or are dead after surviving <5 years. The local SHAP analysis (**a**–**d**) reveals the interaction between B cells (**a**), M0 macrophages (**b**), CD8^+^ T cells (**c**), and NK T cells (**d**) (estimated using EPIC, CIBERSORT, TIMER, and xCell methods) with the ≥5-year survival rates, respectively. Units: i—cell fractions relative to all cells in sample, ii—immune cell fractions relative to total immune cell content, iii and iv—cancer type-specific arbitrary units comparable between samples. Lower SHAP values on the y-axis indicate higher chances of ≥5-year survival in breast cancer patients. The conditional probabilities of ≥5-year survival of all breast cancer patients in various TME conditions from the clinical dataset of patients are shown in (**e**).

Next, we analyzed the influence of the topmost performing TME features (i.e., B cells, CD8^+^ T cells, M0 macrophages, and NK T cells) from the EPIC, CIBERSORT, TIMER, and xCell XAI analyses on the survivability rate in breast cancer patients. In Figure 3, we illustrate the effect of the variations in B cell, CD8^+^ T cell, M0 macrophage, and NK T cell estimates on the models’ predictions to identify the critical inflection points, above or below which the ≥5-year survival rates improve. We found that the B cell fraction > 0.025 (Figure 3a), M0 macrophage fraction < 0.05 (Figure 3b), CD8^+^ T cell fraction > 0.25 (Figure 3c), and NK T cell fraction > 0.075 (Figure 3d) are ideal conditions, as characterized by lower SHAP values on the y-axis, for enhanced ≥5-year survivability chances for breast cancer patients.

We designed seven different TME conditions based on these inflection points:

(i) $C_{1}$: B cells > 0.025,
(ii) $C_{2}$: CD8^+^ T cells > 0.25,
(iii) $C_{3}$: M0 macrophages < 0.05,
(iv) $C_{4}$: NK T cells > 0.075,
(v) $C_{5}$: B cells > 0.025 and M0 macrophages < 0.05,
(vi) $C_{6}$: B cells > 0.025 and CD8^+^ T cells > 0.25, and
(vii) $C_{7}$: B cells > 0.025 and NK T cells > 0.075,

which were coupled with Equation (3) to quantify the conditional probability of ≥5-year survival ($S_{5}$) of breast cancer patients (Figure 3e). We found that the initial probability of ≥5-year survival ($S_{5}$) based on the original dataset was 82.3%. In contrast, the probability of ≥5-year survival ($S_{5}$) given conditions $C_{1}$ to $C_{7}$, i.e., $P(S_{5}|C_{1})$ to $P(S_{5}|C_{7})$, increased by up to ~18% (Figure 3e). In other words, it is possible to increase the ≥5-year survival chances by ~18% by boosting the B cell and CD8^+^ T cell fractions or B cell and NK T cell fractions in the TME above their respective inflection points. The XAI-based revelation of the critical inflection points and the statistical evaluation of certain TME conditions have high potential in deriving novel insights that could have clinical implications for accurate predictions and targeted clinical treatment of patients with breast cancer.

Furthermore, we analyzed the TCGA data to identify the individual TNBC and non-TNBC patients who survived for less than 5 years to verify the effectiveness of the critical TME factors identified by our XAI models on the enhanced survivability rates. We tabulated the individual patients’ clinical data and the associated B cell, CD8^+^ T cell, NK T cell, and M0 macrophage fractions (Table 2 and Table 3). We found that there was only one patient with TNBC (B6-A3ZX) and B cell fraction in the TME above the identified inflection point who survived less than five years after diagnosis. However, this patient was a stage IV TNBC patient, and the effect of higher B cell fraction was likely insignificant on the patient’s survival, as the cancer stage was found in Figure 2e to be the more decisive factor than the B cell count on the cancer prognosis. The anomaly in our identified inflection point for B cells is ~2.8% (one in 36 TNBC and NTNBC patients who survived for less than 5 years). Similarly, we found that the anomaly in our identified inflection point for CD8^+^ T cells is ~8.3%, i.e., three in 36 TNBC and NTNBC patients (AR-A5QQ, BH-A0C1, BH-A1EY) who survived for less than 5 years. The anomalies associated with the M0 macrophage (BH-A1EW, C8-A3M7, EW-A1P8, BH-A0C1, D8-A1Y1, E2-A14Z, LL-A73Z (stage IV patient)) and NK T cells (A2-A0T2 (stage IV patient), AC-A2QJ, B6-A409, EW-A1P8, BH-A18J, E2-A1LE, LL-A73Z (stage IV patient)) inflection points were relatively higher at ~19.4%, i.e., seven in 36 TNBC and NTNBC patients who survived for less than 5 years.

## 4. Discussion

The application of AI models for diagnostic and prognostic assessments has been widely accepted in the context of some cancers [44,45]. The ability of AI models to discover embedded nonlinear patterns within complex multivariate datasets could potentially lead to a better understanding of the complex mechanisms that underlie carcinogenesis and cancer progression [46]. However, recent research indicates that there is a trend toward blind acceptance of black box models that lack transparency and accountability, which could have severe consequences [47]. It is imperative to apply AI models that are inherently interpretable together with XAI methods to produce accurate predictions to better understand the underlying reasoning of the AI approach and discover new interpretable knowledge from large datasets that would otherwise be impossible with traditional statistical techniques [48]. To overcome such problems, which stem from the model’s lack of transparency, we used XAI models comprising tree-based ensembles—which are more interpretable than the black box-type deep learning models (e.g., artificial neural networks)—along with game theory-based explanation models to determine prognoses in patients with breast cancer and to disclose valuable information regarding the ideal TME conditions for improved prognosis and treatments.

In the past, AI analysis has focused on early diagnosis of primary cancers, survival prognosis, or risk of relapse [49,50]. Janizek et al. introduced “TreeCombo,” a gradient of the boosted tree-based approach, in combination with Shapley analysis to predict synergy of novel drug combinations [51]. In various cancers, the density of tumor-infiltrating lymphocytes (e.g., B cells, T cells) correlated positively with survival prognosis [52,53]. Using AI-based analysis (through a random forest tree-based classifier) and CIBERSORT, He et al. [54] reported that high immunity (a subset of TNBC according to the authors) was associated with larger numbers of CD8^+^ T cells, CD4^+^ T cells, NK cells, and M1 macrophages in the TME, and hence was considered to have more favorable clinical outcomes than other subtypes of TNBC. Similarly, with use of AI-based image analysis, He et al. [55] reported that tumor-infiltrating lymphocyte cells were significantly reduced in the TME in metastatic TNBC, compared with the number of these cells in primary TNBC, and larger numbers of tumor-infiltrating lymphocytes were found to be associated with better prognosis.

Our XAI models indicated that boosting the B cell, CD8^+^ T cell, and NK T cell fractions above their inflection points and/or reducing the M0 macrophage fraction to a level below its inflection point in the TME would be conducive for optimal collaboration of the TME features. For example, collaboration between T and B cells to carry out eradication of tumor cells, which may be related to CD4^+^ T cells (identified as the third most influential TME features on breast cancer prognosis from the EPIC, CIBERSORT, TIMER, and xCell XAI analyses in Figure 2) causing B cells to proliferate and their progeny to differentiate into antibody-secreting cells [56]. NK T cells contribute to B cell maturation, antibody and cytokine production, and antigen presentation [57]. Subsequently, the B cells mark the tumor cells for destruction, which is carried out by cytotoxic cells such as CD8^+^ T cells and NK T cells. Furthermore, this response is likely amplified by T cell receptors arming the cytotoxic T cells. To the best of our knowledge, the relative importance and novel interactions between the tumor-infiltrating lymphocytes and tumor-associated cells in the TME on the overall and ≥5-year survival prognosis of breast cancer patients have not been previously reported, although such immune signatures could have potential clinical implications, especially for TNBC treatment.

In previous studies, CD8^+^ T cells were considered to be the crucial effector cells mediating effective antitumor immunity, resulting in better clinical outcomes, whereas intra-tumoral CD4^+^ T cells have negative prognostic effects on breast cancer patient outcomes [43]. Hollern et al. reported that CD4^+^ T-helper follicular cells, B cells, and the antibodies generated by those B cells play important roles in antitumor response to dual immune checkpoint inhibitors in mouse models [58,59]. Furthermore, previous use of rituximab to deplete B cells demonstrated no real clinical benefits for patients with solid tumors [60,61]. NK T cells have a substantial capacity to produce extensive amounts of cytokines upon stimulation to activate NK cells, regulatory and conventional T cells, and B cells [62]. Antitumor efficacy of T cells was shown in vivo to be dependent on the presence of non-T cells, in which the activated NK T cells rendered T cells resistant to myeloid-derived suppressor cells [63]. Considering their broad cytokine profile and potential immune enhancing and immunosuppressive roles, modulating the NK T cells activity towards immune activation has been considered an immunotherapeutic option [64]. Tumor-associated macrophages promote tumor growth by suppressing immunocompetent cells, including neovascularization and supporting cancer stem cells [65], and could also help the tumor cells escape from elimination and spread to other tissues and organs [66]. Macrophages are re-engineered to modulate their regulatory role, for example, to reduce tumor collagen deposition and promote T cell infiltration into breast tumors [67].

The novel insights derived from our XAI model shed light on the strong impacts of B cells, CD8^+^ T cells, and NK T cells, along with M0 macrophages on the survival chances of patients with breast cancer and reveal their critical inflection points for designing innovative strategies to reprogram the TME. The main systemic therapy for metastatic TNBC is chemotherapy, despite the poor prognosis and poor patient survival associated with its use. In such cases, boosting the B cells, CD8^+^ T cells, and NK T cells in the TME as a targeted immunotherapy could serve as a better alternative. Our findings underscore the need to rethink the current cancer immunotherapies focused on harnessing only the antitumor CD8^+^ cytotoxic T cell response. Increased awareness through use of XAI to understand the dynamics of the TME could lead to more rational and evidence-based therapies, leading to improved outcomes in breast cancer patients.

We recognize that one of the major TCGA data analysis results is that at least 80% of the submitted samples must be composed of tumor cells. In the future, the use of other techniques like radiomic analysis to predict histological outcomes, parenchymal enhancement, and single cell proteomics may provide the data required for AI predictions of tumor behavior and outcomes [68,69].

## 5. Conclusions

Our new XAI model framework with enhanced model interpretability and explainability of the results may reduce the concerns about the transparency and accountability of the use of AI models in medical decisions. Our EPIC, CIBERSORT, TIMER, and xCell XAI analyses revealed that B cells, CD8^+^ T cells, NK T cells, and M0 macrophages are the most critical TME features for breast cancer prognosis. Our XAI models further revealed that by boosting the B cell and CD8^+^ T cell fractions or B cell and NK T cell fractions in the TME to levels above their inflection points—identified by XAI analysis in this study—the survival rate of breast cancer patients could increase by up to 18%. They could be alternative immunotherapies to conventional breast cancer therapeutics, although these findings require further in vitro and in vivo testing and clinical verifications.

## Figures and Tables

**Table 1:** Statistical validation of the predictive accuracies of the AI models.

TME Immune Cell Estimation Method	Accuracy (%)	Precision (%)	Recall (%)	F1 Score (%)
EPIC	96.4	93.6	100.0	96.7
CIBERSORT	98.8	97.8	100.0	98.9
TIMER	95.2	91.7	100.0	95.7
xCell	100.0	100.0	100.0	100.0

**Table 2:** Clinical and critical immune cell composition (identified via XAI) from bulk RNA-seq data of TNBC patients that survived for less than 5 years.

TCGA Patient ID	Stage	Age	Months	B Cell	M0 Macrophage	CD8^+^ T Cell	NK T Cell
A1-A0SK	II	54	31.8	0.003	0.325	0.000	0.000
A2-A0CM	II	40	24.8	0.008	0.129	0.025	0.013
A2-A0T2	IV	66	8.4	0.001	0.077	0.098	0.183
A2-A3XY	II	49	35.9	0.013	0.261	0.049	0.072
AC-A2QJ	III	48	14.7	0.000	0.196	0.000	0.146
AR-A5QQ	III	68	10.6	0.017	0.109	0.259	0.061
B6-A3ZX	IV	50	37.9	0.145	0.058	0.110	0.058
B6-A409	III	44	18.8	0.002	0.052	0.000	0.096
BH-A1EW	II	38	55.7	0.004	0.000	0.244	0.047
C8-A3M7	III	60	34.0	0.008	0.000	0.216	0.014
E2-A1LK	III	84	8.7	0.005	0.650	0.000	0.034
EW-A1P8	III	58	7.9	0.003	0.000	0.034	0.101

**Table 3:** Clinical and critical immune cell composition (identified via XAI) from bulk RNA-seq data of non-TNBC patients that survived for less than 5 years.

TCGA Patient ID	Stage	Age	Months	B cell	M0 Macrophage	CD8^+^ T Cell	NK T Cell
A2-A0SV	IV	63	27.1	0.000	0.173	0.016	0.038
A7-A13E	II	62	20.2	0.001	0.504	0.118	0.005
A8-A08J	IV	52	37.1	0.003	0.240	0.014	0.050
AC-A23H	II	90	5.7	0.002	0.419	0.150	0.003
AR-A0TY	II	54	55.9	0.007	0.270	0.030	0.043
BH-A0C1	III	61	46.4	0.002	0.000	0.284	0.034
BH-A18J	IV	56	20.1	0.001	0.375	0.084	0.081
BH-A18P	I	60	30.3	0.003	0.159	0.230	0.049
BH-A18T	II	70	7.4	0.001	0.157	0.000	0.008
BH-A1EV	III	45	12.0	0.001	0.310	0.087	0.053
BH-A1EX	II	67	49.6	0.003	0.061	0.147	0.035
BH-A1EY	II	79	17.7	0.001	0.072	0.250	0.025
BH-A1F8	III	90	25.1	0.005	0.069	0.188	0.000
BH-A1FD	I	68	33.2	0.000	0.130	0.051	0.004
C8-A12Q	III	78	12.7	0.007	0.264	0.177	0.038
D8-A1XC	III	85	12.4	0.004	0.266	0.126	0.041
D8-A1Y1	III	80	9.9	0.000	0.000	0.050	0.010
D8-A73W	III	79	12.7	0.002	0.290	0.000	0.072
E2-A14Z	I	64	18.5	0.005	0.018	0.147	0.067
E2-A1LE	III	71	28.9	0.002	0.173	0.219	0.085
E9-A1N6	II	52	22.3	0.000	0.313	0.081	0.038
E9-A1NF	II	60	35.2	0.000	0.280	0.124	0.033
LL-A73Z	IV	55	7.5	0.006	0.045	0.130	0.104
UU-A93S	IV	63	3.8	0.001	0.275	0.038	0.069

## Data Availability

The data and models will be made available upon request.
